# COVID-19 Biomarkers and Advanced Sensing Technologies for Point-of-Care (POC) Diagnosis

**DOI:** 10.3390/bioengineering8070098

**Published:** 2021-07-12

**Authors:** Ernst Emmanuel Etienne, Bharath Babu Nunna, Niladri Talukder, Yudong Wang, Eon Soo Lee

**Affiliations:** 1 Advanced Energy Systems and Microdevices Laboratory, Department of Mechanical and Industrial Engineering, New Jersey Institute of Technology, Newark, NJ 07102, USA; eee9@njit.edu (E.E.E.); bn63@njit.edu (B.B.N.); nt22@njit.edu (N.T.); yw35@njit.edu (Y.W.); 2 Division of Engineering in Medicine, Department of Medicine, Brigham, and Women’s Hospital, Harvard Medical School, Harvard University, Cambridge, MA 02139, USA

**Keywords:** COVID-19 biomarkers, COVID-19 sensing techniques, biomarker detection, biomarker sensing, future diagnostic trends

## Abstract

COVID-19, also known as SARS-CoV-2 is a novel, respiratory virus currently plaguing humanity. Genetically, at its core, it is a single-strand positive-sense RNA virus. It is a beta-type Coronavirus and is distinct in its structure and binding mechanism compared to other types of coronaviruses. Testing for the virus remains a challenge due to the small market available for at-home detection. Currently, there are three main types of tests for biomarker detection: viral, antigen and antibody. Reverse Transcription-Polymerase Chain Reaction (RT-PCR) remains the gold standard for viral testing. However, the lack of quantitative detection and turnaround time for results are drawbacks. This manuscript focuses on recent advances in COVID-19 detection that have lower limits of detection and faster response times than RT-PCR testing. The advancements in sensing platforms have amplified the detection levels and provided real-time results for SARS-CoV-2 spike protein detection with limits as low as 1 fg/mL in the Graphene Field Effect Transistor (FET) sensor. Additionally, using multiple biomarkers, detection levels can achieve a specificity and sensitivity level comparable to that of PCR testing. Proper biomarker selection coupled with nano sensing detection platforms are key in the widespread use of Point of Care (POC) diagnosis in COVID-19 detection.

## 1. Introduction

### 1.1. COVID-19 History and Statistics

COVID-19 has become the fifth documented pandemic since the 1918 flu [1]. Due to the disease’s speed and fast scale transmission, WHO (World Health Organization) declared the virus a pandemic in March 2020 [2]. The International Committee on Taxonomy officially named the virus Severe Acute Respiratory Syndrome Coronavirus 2 (SARS-CoV-2) [3]. While the two names are synonymous, COVID-19 is widely used. Around the world, death tolls have surged, and according to the Centers for Disease Control (CDC), this virus has claimed the lives of more than 2,915,972 people and counting [4,5]. COVID-19 was first detected in a seafood market that sells live animals on 8 December 2019. The market was in Wuhan, China, in the province of Hubei [6]. At the onset, 27 patients experienced pneumonia and were all linked to the market; within a few weeks, the virus spread rampantly through China [7]. After a month, the spread reached several countries, including Italy, the United States, and Germany. The rate of the spread could be directly correlated to the population density of Wuhan. There are more than 9 million full-time and 5.1 million transient residents in this region, respectively [8]. The pandemic impacted countries economically as many large-scale businesses and companies experienced substantial financial loss. The world economy plummeted, and countries experienced a recession similar to 1870 [9].

According to early research, scientists have linked the virus’s origins to bats. The virus was then transmitted from its source to an intermediate host, possibly Pangolin, followed by human-to-human transmission. As the evidence supporting this claim increases, currently, the possibility of other routes of transmission should not be ruled out [10].

Once an individual is infected, there must be an efficient way to detect the virus. Biomarkers help to enhance the development and approval of new innovative drugs and biological products in the field of vaccines. They also help predict future complications or severity of disease and serve as indicators for COVID-19 prognosis and diagnosis [11].

### 1.2. Importance of COVID-19 Biomarkers in Virus Detection and Sensing

**Figure 1:**
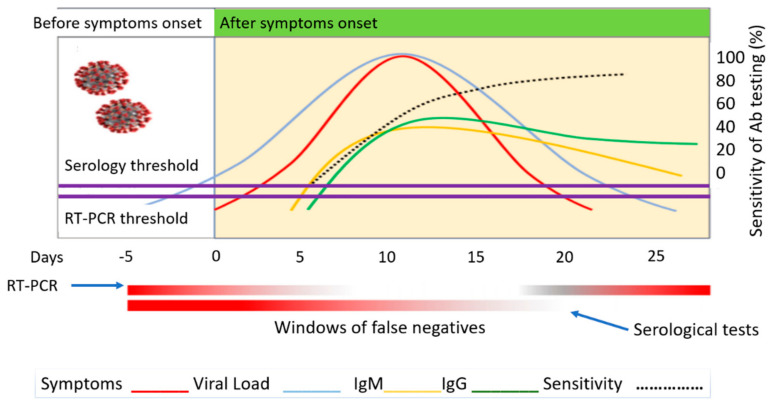
COVID-19 biomarker detection levels over time.

At the onset of patient exposure to the virus, there is usually a time lag when symptoms begin to develop. This lag is approximately 5 to 7 days. During this time, an RT-PCR test would reveal false-negative results. The viral load is too low, and it increases through replication in the body. More cells are infected, and symptoms start to develop. Symptoms typically follow the same trend as the viral load. Once the viral loads reach 100 copies of Viral RNA per ml the RT-PCR threshold has been reached. Immunoglobulins response levels increase as a response to this viral load. Depending on the individual, seroconversion can be detected as early as 5 to 7 and 14 days after symptoms develop. As a patient begins to recover and viral loads start to decrease, immunoglobulin IgM levels increase until about 10 days after symptom onset. Afterward, IgM levels drop off rapidly as compared to IgG which tends to stabilize after 25 days. This means that depending on the individual, the first two weeks are usually the most opportune time to perform viral testing using RT-PCR and the least opportune time to perform serological testing for immunoglobulin detection. Understanding the reason for the variation will minimize false-negative testing. Sensitivity is the ratio of the true positive test and the total of individuals with the disease. The sensitivity of RT-PCR and serological tests are based on this same concept. The detection of antibodies starts at a minimum of 5 days after symptom onset and peaks 25 days when the highest concentration of IgG antibodies is detected. The dotted black line in Figure 1 reported by LaMarca et al. [12] also shows the sensitivity rise over time of the chemiluminescent assay as derived from the datasheet of a commercial test (Abbott Diagnostics, Lake Forest, Ill.USA). The percentages are reported as (53.1%) after day 7, (82.4%) after day 10, (96.9%) after 14 days and (100%) after 17 days. The highest chance of antibody detection in an infected patient is after 17 days.

**Figure 2:**
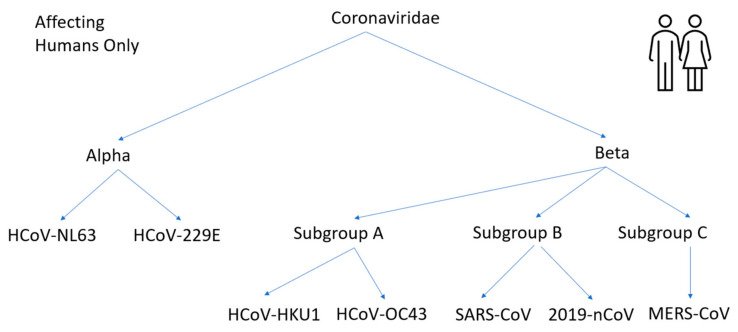
Coronaviruses affecting the Human Body.

During testing for the virus, the window of false-negatives must be acknowledged and determined beforehand to ensure tests are valid. It is also true that for rapidly transmitted diseases, such as COVID-19, with no cure available, the most effective way to curb the spread is through early detection and isolation of those infected [13]. Historically, there are several types of coronaviruses that have been discovered. The virus received the moniker because of the crown-like glycoprotein spikes found on their surface [14]. Coronaviruses are enveloped, positive-sensed single-stranded (Ribonucleic Acid) RNA Virus [15]. The four major categories of coronaviruses are alpha, beta, delta, and gamma. Delta and gamma groups affect mostly avian species, while alpha and beta primarily affect mammals [16]. The scope of this paper focuses on the viruses that affect humans. This is illustrated in Figure 2 adapted from [17].

Bats are the natural hosts of alpha and beta coronaviruses [18]. In the alpha group and beta group, HCoV-NL63, HCoV-229E, HCoV-HKU1, HCoV-OC43 generally cause mild to moderate upper respiratory tract illness and contribute to 15–30% of cases and are referred to as common cold coronaviruses [19]. SARS-CoV, MERS-CoV, and 2019-nCoV differ from mild forms of HCoV viruses described previously because of their severity once contracted and the higher recorded death rates in patients. SARS-CoV and 2019-nCoV belong to the same beta subgroup b and bind to (angiotensin-converting enzyme 2) ACE2 receptor and use (Transmembrane Serine Proteases) TMPRSS to cleave both the S (spike) protein and ACE2 receptor to promote viral spread. The 2019-nCoV binds to the receptor with high binding affinity [20,21]. All the viruses differ in their receptor binding characteristics and interactions. On a molecular level, there are variations in their spike protein structure. SARS-CoV-2 shares 79% genome sequence identity with SARS-CoV and 50% with MERS-COV. MERS-CoV binds to a different cell receptor called DDP4 (dipeptidyl peptidase 4) expressed in the human respiratory tract. Furthermore, MERS-CoV was transmitted to humans from camels, a different intermediary host species, while SARS-CoV was transmitted to humans from a palm civet [22].

On 27 March 2020, the FDA (Federal Drug Administration) issued an (Emergency Use Authorization) EUA because COVID-19 was declared a public health emergency. The issuance of the authorization helped allow more timely access to critical medical products such as medicines and tests when there were not many approved and available options [23]. As a result, many new sensing technologies and detection mechanisms for COVID-19 biomarkers were developed. Currently, the gold standard for detection is the (Reverse Transcription-Polymerase Chain Reaction) RT-PCR. It has one of the highest accuracies in virus confirmation. However, the early form of this laboratory testing has several limitations. It requires trained personnel and costs over USD 100 (United States Dollars) for a single kit. The setup costs more than USD 15,000, and the analysis time is 4 to 6 h while taking over one day to receive results [24]. As the number of infected grew, this established a growing need to reduce the time it took to obtain results and increase the availability of tests. This need was satisfied by the advent of Point of Care (POC) technologies. They allow physicians to detect and diagnose diseases near a patient site as opposed to the lab. These tests typically have a rapid turnaround time of approximately 15 min. They are low-cost and easy to use.

**Figure 3:**
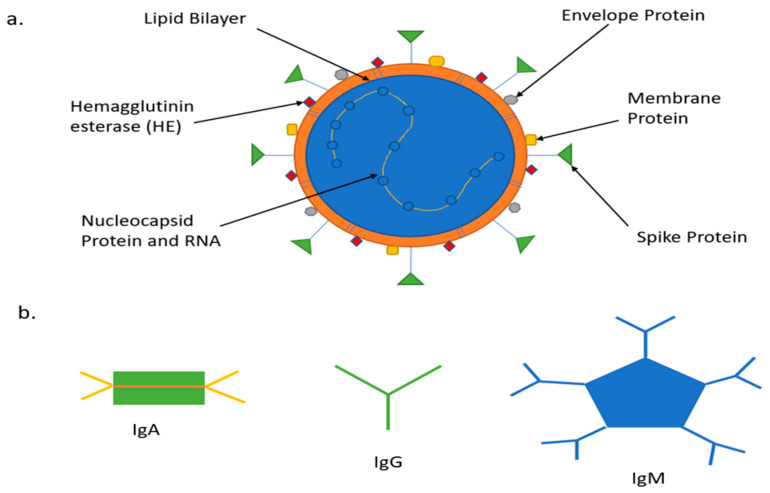
COVID-19 biomarkers (**a**) Schematic representation of COVID-19 virus; (**b**) Schematic representation of COVID-19 antibodies.

In the past, POC technologies assisted in glucose monitoring, pregnancy, infertility testing, infectious disease testing, cholesterol testing, cardiac monitoring, and many other forms of testing [25,26,27,28,29]. The focus of this paper will be COVID-19 sensing mechanism platforms for different biomarker detection, including Nucleic Acid Amplification for Viral RNA, Antigen Testing Nucleocapsid (N), Spike (S), Protein and Serological Test for antibodies (Immunoglobin) IgA, IgM, IgG and Interleukin (IL-6) inflammatory biomarkers. Figure 3 is adapted from [30].

## 2. COVID-19 Biomarkers Classification

### 2.1. Genome Structure and Antigens

The virus is a single-strand positive-sense RNA (+ssRNA), ranging from 27–32 kilobase pairs (kbp). Genomic 5′ is the leading end of the structure and 3′ is the tail end. The genome consists of two large genes Open Reading Frame (ORF1a and ORF1b), which encode nonstructural proteins (NSP 1–16) and form the replicase/transcriptase complex. These two genes combine to make two-thirds of the genome [31]. These encode for the papain-like protease, 3CL-protease, RNA dependent RNA polymerase (RdRp) and the helicase. The structural gene unit encodes proteins such as spike (S), envelope (E), membrane (M), nucleocapsid (N), and accessory proteins which include hemagglutinin esterase (HE). The S protein is responsible for recognizing host cell receptors, M protein shapes the virus, E protein is responsible for virions assembly and release, and consists of the lipid bilayer. The N proteins are involved in packaging the RNA genome [32]. Furthermore, the S protein is divided into two subregions S1 and S2. The N terminal region of S1 contains the receptor-binding domain (RBD), which detects the angiotensin ACE2 receptor, and the C terminal S2 is responsible for the viral and cellular membrane fusion. The RBD for the virus has a unique feature. It can alternate between an upright position for receptor binding and recline for immune evasion. This contributes to the rapid spread, severe symptoms, and high death rates of COVID-19 [33]. SARS-CoV-2 S binds to human ACE2 with a dissociation constant (KD) of 14.7 nano Molar(nM), while SARS-CoV S binds at 325.8nM indicating that SARS-CoV-2 S has higher sensitivity to ACE2 than is SARS-CoV S [34]. Researchers determined that the furin cleavage site of SARS-CoV-2 is unique and not found in other SARS-like Coronaviruses.SARS-CoV-2 S has multiple furin cleavage sites, which boots its infectivity. S protein is the target protein for pairing with antibodies. Different coronaviruses use distinct domains within the S1 subunit to recognize a variety of attachment and entry receptors, depending on the viral species. The S protein is exposed on the surface and facilitates entry into host cells. It is the main target of neutralizing antibodies (Abs) upon infection and the focus of therapeutic and vaccine design. However, other proteins are used to increase the sensitivity and specificity of the test. In a recent research study conducted by Shah et al. [35], four different proteins of the COVID-19 virus were used to detect which pairing had the greatest affinity with IgM and IgG antibodies over time. A total of 231 patients with various ailments were used in the study. The antigens used were S1, S2, N and RBD. The results help to determine that S2 had the highest overall pairing in the immunoblot assay with IgG and IgM followed by N, S1 and RBD, respectively [36,37].

### 2.2. COVID-19 Antibodies

**Figure 4:**
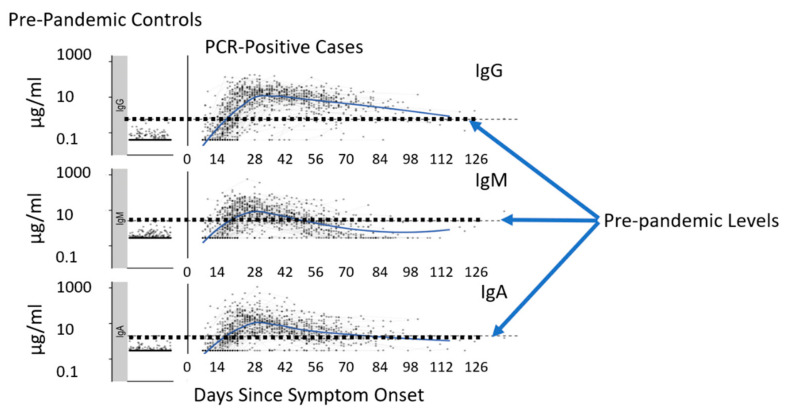
COVID-19 pre-pandemic compared with pandemic IgG, IgM and IgA antibody levels.

Upon infection, the human body develops responses to foreign bodies or invaders. Five prominent antibodies that respond in times of distress are: IgA, IgG, IgM, IgD, IgE. These are called Ig (immunoglobulin) and are Y-shaped proteins made by the immune system B lymphocytes or B cells [38]. IgG has four subclasses or Isotypes, Ig1, Ig2, Ig3 and Ig4. Ig1 has the greatest affinity to the spike protein and is the most dominant IgG subclass out of these four subclasses [39]. A study was conducted by researchers led by Richelle Charles, Massachusetts General Hospital in Boston, MA. They compared blood samples over 122 days of 343 patients to 1500 samples taken before the pandemic began. They identified that out of the five biomarkers mentioned above, only three of these biomarkers IgG, IgM, IgA had detectable concentration levels [40]. These antibodies lock on to their specific antigens and block them from binding to the host cell. At the onset of a COVID-19 infection, IgA and IgM are the first responders. They can be detected as early as four days after symptoms develop. IgG is the most prevalent circulating immunoglobulin and develops a few days after IgM. It has a lasting effect against immunity as its presence can be detected in serum for weeks, months and even years, depending on the individual. IgA protects mucosal surfaces against pathogens and is secreted in human saliva [41]. As time elapses, IgA and IgM concentration increases but then decreases as they seem to be ineffective in binding to the spike protein of the coronavirus. The IgG antibodies seem to have higher success in binding to the antigen [42]. Measurements of IgG, IgM, IgA against SARS-CoV-2 spike protein receptor-binding domain were compared among pre-pandemic controls and PCR positive cases. Concentrations for the following biomarkers were determined during the pre-pandemic period as a form of control IgG: 0.57 microgram/milliliter (µg/mL), IgM: 2.63 µg/mL, and IgA: 2.02 µg/mL and values > 10 µg/mL for patients with a potential positive PCR case. Shown in Figure 4 [43,44].

In terms of tracking disease progression, one study conducted with 105 COVID-19 patients and non-patients showed a peak with IgM at 15–21 days and then dropped while IgG peaked between 22–39 days and lasted for a longer time. In non-ICU patients, N-IgM (N protein to IgM binding) shared a similar profile to N-IgG in the first two weeks from a sample taken from one patient. The level of IgM increased up until the third week, and IgG surpassed it. S-IgG levels were much higher than any other antigen and antibody pairing and showed more promise in protecting patients and have a longer-lasting effect towards immunity [45]. In severe cases of COVID-19 inflammatory biomarkers such as IL-6 found in serum can also be present, which tend to develop because of the disease [46].

### 2.3. Biomarker Progression in the Human Body

COVID-19 can make its way into the human body through contact transmission, droplet transmission, and airborne transmission [47]. At the onset, the virus enters the body through inhalation of aerosols respiratory droplets. These bind to the nasal epithelial cells, which have the ACE2 receptor in the upper respiratory tract [48]. At this point, the individual becomes highly contagious, and a nasal swab can detect both Viral RNA and Antigen biomarker remnants of the virus. Chemokine ligand (CXCL) 10 and interferons (IFN- beta and IFN-lambda) are released as an early response [49]. For the more fatal cases, the virus travels through the airways and makes its way to the upper respiratory system. As the virus replicates and infects more epithelial cells, many cytokines and interleukins known as inflammatory biomarkers are released by the macrophage [50]. Patients with a mild or moderate form of the virus had low levels of inflammatory cytokines and higher levels of Epidermal Growth Factor (EGF), Platelet-derived Growth Factor (PDGF), and Vascular Endothelial Growth Factor (VEGF) [51]. However, patients who experienced a severe form of the virus or died expressed elevated pro-inflammatory chemokines and cytokines, including CXCL10, CCL3, IL-1alpha, IL-1beta, IL-6 IL-18, and TNF-alpha [52,53]. This is more commonly referred to as cytokine storm syndrome [54,55]. In one study, Interleukin 6 (IL-6) biomarker levels greater than 80 picogram/milliliter (pg/mL), C-reactive protein level > 97 milligram/milliliter (mg/mL), IL-1beta > 0.064 pg/mL, and TNF-alpha > 2.23 pg/mL predict a greater risk of respiratory failure [56]. The ACE2 receptor is found in many organs throughout the human body and can perhaps provide a link as to why the virus can spread to other organs besides the lungs [57]. Table 1 summarizes inflammatory biomarkers levels detected in adult patients with COVID-19 [58].

## 3. Advanced Sensing Technologies and COVID-19 Biomarker Detection

### 3.1. Specimen and Sample Types

**Figure 5:**
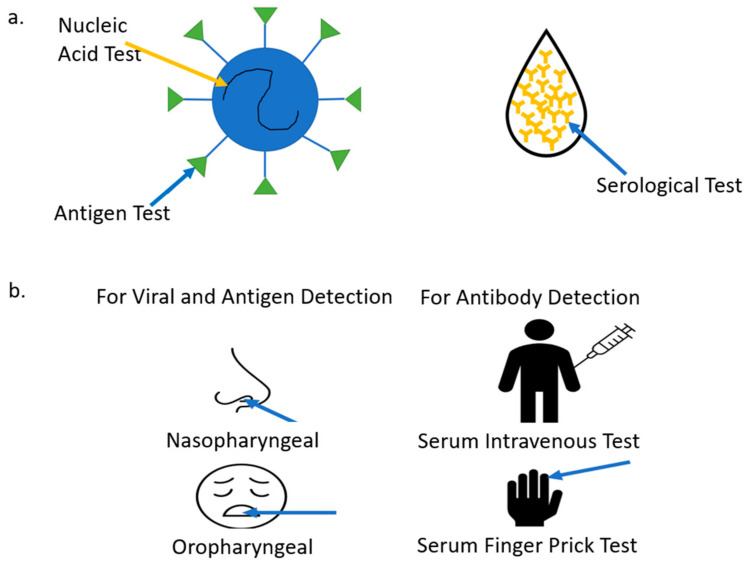
COVID-19 biomarker detection tests (**a**). Types of COVID-19 tests; (**b**) How to extract biomarkers.

There are three main types of COVID-19 tests: antigen, nucleic acid, and serological. The antigen test detects proteins that make up the virus. The N and S protein are the most tested. The nucleic acid test extracts the viral RNA from the virus, converts it to complementary DNA (cDNA), and uses primer to target specific genes of the virus to amplify and replicate. These two tests detect if an individual is currently infected with COVID-19, and biomarkers must be extracted from the respiratory tract. The serological test detects past infections and checks for an immune response through immunoglobulins detected in the blood [59]. Viral RNA and antigen extraction from a patient is performed nasopharyngeal or oropharyngeal. The extraction is performed through a swab and will test a person for a COVID-19 infection. Once a swab is inserted into the nasal cavity, it should go back a distance equal to that of the nostrils to the outer opening of the ear [60]. The swab should pass the anterior portion of the nasal cavity. The CDC recommends that the swab remains in place and rotated to absorb secretions. This will ensure that adequate viral biomarkers are extracted for testing purposes. For oropharyngeal swabbing, the focus should be directed towards the rear wall of the oropharynx and rotated before removal [61]. A study conducted with 353 patients revealed that a higher positive rate for SARS-CoV-2 was detected using nasopharyngeal extraction [62]. Not only can nasal and oral swabs be used for viral detection, but new research also shows that they can also be used to detect antibodies. However, the concentration levels are low, requiring a high-level detection device. Another study showed that during a three-month period where antibodies were extracted from nasal or oral swabs, there was no decrease in IgG levels as compared to that of serum. The levels were consistent in antispike IgG, anti RBD IgG and anti NP IgG. Levels for both IgA and IgM did decline however, as expected. Out of the three isotypes, IgA showed the least correlation since IgA levels are indeed higher in saliva. If antibodies are detected in saliva, it would be reasonable to assume that plasma cells that release the antibodies migrate to these areas during the infection period. Oral swabs seem to be a more preferred biomarker extraction since they are, in fact, the least invasive [63]. Serological testing is one of the premier ways to extract antibodies. These biomarkers are detected in blood, plasma, or serum. Blood plasma contains white blood cells, red blood cells and platelets. The serum is the fluid that remains after removing fibrinogen, also referred to as clotting agents. The serum is harder to obtain because it needs additional separation [64,65,66,67]. Sample extraction is not limited to the options mentioned above. In several studies, fecal samples are used to detect viral RNA. Viral RNA concentrations from fecal specimens were shown to last longer than that of the respiratory tract. In some instances, they can be detected nearly five weeks after a patient’s respiratory sample tests negative [68]. Figure 5a shows the three main types of COVID-19 tests adapted from [69]. Figure 5b provides different methods for biomarker extraction adapted from [70].

### 3.2. Currently Implemented Tests

#### 3.2.1. Reverse Transcription-Quantitative Polymerase Chain Reaction (RT-PCR)

Nucleic acid amplification testing or (NAAT) is one of the primary methods used to diagnose and detect COVID-19. This process essentially involves the reverse transcription of viral RNA into complementary DNA through RT-PCR [71,72,73,74]. Then complete specific regions of the complimentary Deoxyribonucleic acid (cDNA) are amplified for further detection using fluorescent dye or electrical signals. Sequence targeting agents or primers help identify and confirm a patient’s SARS-CoV-2 infection status. The critical components of NAAT are sequence alignment, primer design, assay optimization, and testing. Many molecular diagnostic tests have used real-time RT-PCR technology to target various genomic regions of SARS-CoV-2. These regions include the ORF1b or ORF8 regions, the nucleocapsid (N), spike (S) protein, RNA-dependent RNA polymerase (RdRP), or envelope (E) genes [75,76,77,78]. In a research study, components of the SARS viral genome were aligned and analyzed to determine the best set of primers and probes. The results helped to determine three regions that had conserved sequences RdRP gene in the ORF1ab region, the E gene, and the N gene. The RdRP and E genes had a technical limit of detection of 3.6 and 3.9 copies per reaction, respectively. The N gene provided a worse technical limit of detection at 8.3 copies per reaction [79]. RT-PCR is usually a one-step or two-step procedure. The one-step procedure uses a single tube with the primers to complete the reaction. The two-step procedure uses more than one tube to complete transcription and amplification reactions [80,81,82]. Additionally, the latter has higher flexibility and sensitivity. However, the one-step procedure is quick to set up and minimizes the chances of cross-contamination. In advanced detection methods, such GenMark eSensor, target DNA is mixed with ferrocene labeled signal probes that pair with specific DNA targets. The probes are bound to gold-plated electrodes, which generate specific electrical signals measured with voltammetry [83].

#### 3.2.2. Reverse Transcription-Loop Mediated Isothermal Amplification (RT-LAMP)

**Figure 6:**
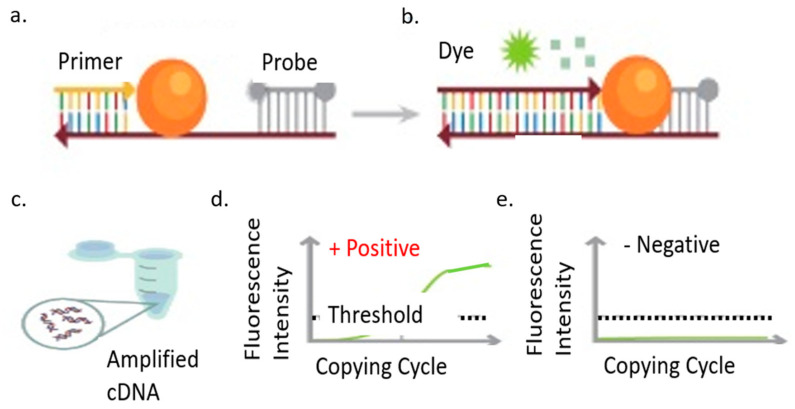
COVID-19 nucleic acid amplification RT-PCR test, (**a**). Primer application, (**b**). Fluorescent dye used for visual amplification, (**c**). Complimentary DNA amplified through RT-PCR process, (**d**). Positive case threshold surpassed, (**e**). Negative case threshold not reached.

**Figure 7:**
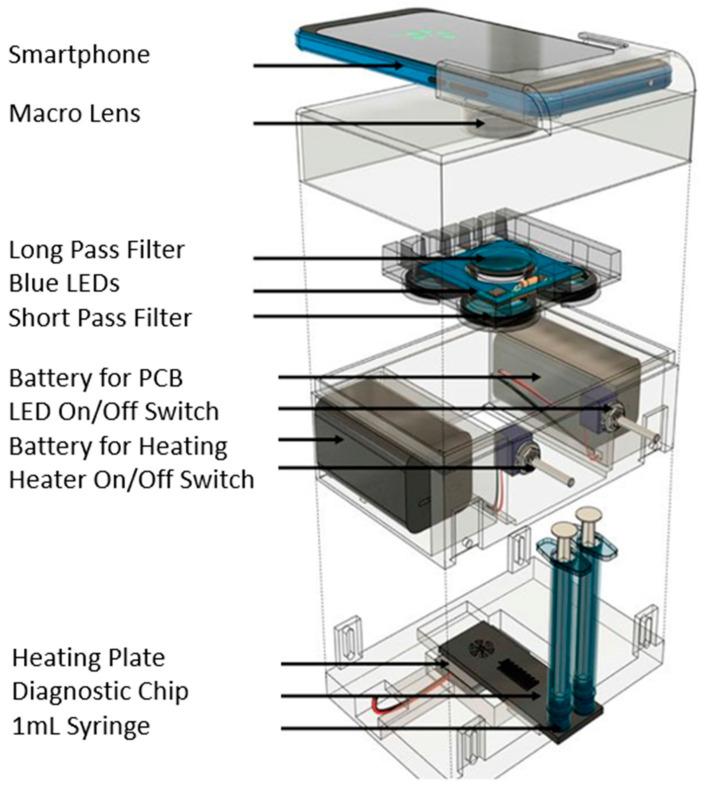
COVID-19 nucleic acid amplification RT-LAMP test.

Loop-mediated isothermal amplification (LAMP) is a recent rapid technology of DNA amplification. Compared to a PCR test that requires temperature cycling, the LAMP reaction occurs at a constant temperature of 65 °C [71,72,73,74,75,76,84,85,86,87]. Furthermore, the target DNA amplifies in only 30 min. The LAMP method employs 4 or 6 primers to bind six regions of a target DNA with high specificity. Combining RT and LAMP shortens the reaction time as a purification step is not required [88,89,90,91,92]. The detection limit is 80 copies of viral RNA per ml sample, and results can be observed through a smartphone or lens. Typically, a fluorescent dye helps to highlight the viral amplification process as it occurs. A recent new technology was combined with RT-LAMP to increase the accuracy of COVID-19 biomarker detection. CRISPR/Cas12a-based precision technique, called SARS-CoV-2 DNA endonuclease-targeted CRISPR trans reporter (DETECTR), allows for the diagnosis of COVID-19 RNA extracted from swab samples in less than 40 min. The integration of the CRISPR/Cas12a DETECTR system contributes to the quick detection process. Cas12a detects predetermined viral sequences, and the reporter molecule’s cleavage confirms the presence of a virus. Compared to RT-PCR, this detection method eliminates the need for a slow turnaround time, target specificity for single nucleotides, integration with lateral flow strips and other POCs, and no requirement for sophisticated laboratory systems. This system is a high-speed and a visual alternative to RT-PCR for detecting SARS-CoV-2 with 95% and 100% agreement for positive and negative prediction, respectively [93,94,95,96]. The importance of primer selection for RT-LAMP cannot be understated to help in the rapid detection of SARS-CoV-2 in infected patients [97,98,99,100]. The N1 primer set had a sensitivity of two RNA copies per reaction of 25 µL in 20 min of reaction time. The N15 and S17 primers have the same detection limit of two RNA copies per reaction but thirty minutes of reaction time. The O117 primer set could only detect 20 RNA copies per reaction in 30 min. The N1 primer set had the best performance. If RNA extraction is not required for this assay, it is essential to investigate its effect on results. As observed, after forty minutes of the detection process, a color change was shown, without the RNA extraction step. The N15 and O117 primer set accurately detected all eight positive and negative clinical samples [101]. Figure 6 identifies the main process of RT-PCR amplification [102] and Figure 7 a portable RT-LAMP amplification apparatus [103].

### 3.3. Recent Advances in COVID-19 Sensing

Sensors are made from chemical or biological receptors and transducers. The receptor detects and interacts with the analyte and the transducer converts it into a recognizable signal [104,105,106,107]. Biosensors use enzymes, antibodies, or nucleic acids in tandem with a transducer and detector to provide output. There are 11 features that an ideal biosensor should have which are: multiple sensing modes, high sensitivity, quick response time, multiplexing capabilities, disposable, long shelf life, easy to use, cost-effective, mass manufacturable and autonomous [108,109,110,111]. Incorporating these features in new technologies will help improve the sensing or detecting platforms of the future.

#### 3.3.1. Optical Biosensor

Optical biosensors are an alternative method for COVID-19 virus detection. A few characteristics of this detection platform include excellent sensitivity levels, minimizing interferences due to electromagnetic disturbances, and ease of use. Their detection capabilities are preferred due to their safe, ease of use, and cost-effective technology [112]. Optical biosensors measure changes in the optical properties of the propagated light when a binding interaction occurs between the immobilized receptor, most times an antibody or antigen with the target analyte. There are many different types of optical sensors. Among them are photonic and plasmonic biosensors, which have high sensitivity and do not require amplification as the RT-LAMP and RT-PCR [113,114,115]. The detection process starts with an evanescent wave forming as light travels in a medium and undergoes internal reflection (TIR). The wave penetrates the surrounding dielectric medium and reduces in intensity. The evanescent field detects the changes in the refractive index (RI) of the medium and alters the light properties that vary in intensity, phase, resonance momentum, or polarization [116,117]. The SPR biosensor generally employs a 40 to 50 nanometer (nm) layer of gold as a transducer. An incident light beam excites the electrons along the interface metal-dielectric, generating an evanescent field that can extend 10 to 300 nm into the surrounding medium. When specific bioreceptor elements are immobilized onto the gold surface, the selective capture and binding of the target molecule change the refractive index and light properties. This change is directly proportional to the analyte concentration in the sample. The RI limit of detection of SPR biosensors typically reaches 0.00001–0.0000001 refractive index units (RIU), which commonly relates to detection limits in the low nM or even pM level in surface analyte detection. Silicon photonics technologies have arisen as leading platforms in sensitivity and integration capabilities. Silicon photonics biosensors are fabricated on Silicon substrate materials. Light travels and generates an evanescent electromagnetic field with penetration depths between 100 and 900 nm and is used as a probe. The optical sensing technology coupled with a low power requirement and the capability to incorporate multifunctional capabilities (chemical, optical, microfluidics, and electronics) on one platform help advance them in POC. Furthermore, optical biosensors can increase the multiplexing capability. They can detect various biomarkers of a virus all at once through multiple channels on the same chip [118,119]. Localized surface plasmon resonance (LSPR) is based on strong photon-driven coherent oscillation of the surface conduction electrons. This dual-functional plasmonic biosensing concept integrated the plasmonic photothermal PPT effect, due to absorbed light being converted to heat energy, and the LSPR sensing transduction on a single Au-Gold Nano Island (AuNI) chip. The resonances of PPT and LSPR are excited two different wavelengths at different angles of incidences which enhance detection results of various viral sequences Applying two different angles of incidence, the plasmonic resonances of PPT and LSPR can be excited at two different wavelengths, which significantly enhanced the sensing stability, sensitivity, and reliability. With this configuration, the LSPR sensing unit attained a real-time and label-free detection of viral sequences including RdRp-COVID, ORF1ab-COVID, and E genes from SARS-CoV-2. Thus, based on the LSPR signal target size relationship, the estimated LOD for detecting the entire RNA strands from SARS-CoV-2 could be approximately 2.26 × 10^4^ copies. A recent study reported the viral loads of SARS-CoV-2 from different respiratory trace samples including the throat/nasal swabs and the sputum. However, there are current limitations that prevent widespread use of this technology for COVID-19 POC which include difficulties in obtaining a suitable substrate and reusability [120,121,122].

#### 3.3.2. Piezoelectric Biosensor

A piezoelectric-based sensor produces a voltage when under mechanical stress. An anisotropic crystal is used for the detection of oscillations because of its unique properties. The sensor is activated by an alternating voltage at the electrodes, which propagate to the surface. The analyte is deposited on the crystal, and the frequency shift is measured [123,124]. When molecules interact, mass (m) increases due to the interactions between molecules, the frequency (f) controlled by the AC voltage decreases. Mass response-type piezoelectric sensors are standard for virus detection. This sensor can use both antigen and antibody biomarkers for detection. Probe antibodies are placed on the upper electrode surface. The upper and lower electrodes drive the resonation of the piezoelectric material. The target antigen then binds with the probe antibodies. The mass change on the electrode surface creates a frequency shift of the material in the oscillation circuit which can be measured [125,126,127,128]. The major drawback of this detection method is its limitation on the size as it typically works for high molecular weight analytes since it reduces the oscillation frequency. Various types of anisotropic materials help in sensing, for example, aluminum nitride, zinc oxide, barium titanate, lead titanate, quartz, and poly (vinylidene fluoride) [129,130]. The first piezoelectric immunosensor was able to detect the coronavirus in sputum. On the surface of the Piezoelectric Quartz Crystal (PQC), horse polyclonal antibodies were immobilized for antigen detection. The sensor achieved antigen detection by spraying a form of aerosol powder dissolved in the sputum of a non-infected person. Through ultrasonic oscillation, a frequency shift was detected and verified to be proportional to the antigen concentration range of 0.6–4 µg/mL. The sensor performed very well and was reused over 100 times without any significant issues as long the storage temperatures were maintained at 4–6 degrees Celsius [131,132,133].

#### 3.3.3. Electrochemical Biosensor

The electrochemical biosensor uses biochemical reactions that are converted into electrical signals that can be detected as amperometric, potentiometric, impedimetric, or conductometric [134,135,136]. Electrochemical biosensors usually consist of working, counter, and reference electrodes and allows for the unique detectable pairing of the antibody and antigen. Electrochemical platforms can use gold nanoparticles, deposited on a Titanium surface as a sensing electrode. This layer has high stability and is immune to chemical treatments and processes. Gold is a noble metal with high thermal and electrical conductivity, which helps with the sensing process [137,138,139,140]. In more advanced sensing, carbon or metallic nanostructures are used to increase sensitivity by enhancing immobilization of the biomarkers or binding of target molecules due to increased surface area. Paper-based electrochemical sensing is a newer type of sensing using a wax printer. It consists of three folding layers and three electrodes. Spike protein is immobilized on the test zone of the working pad with the aid of various chemicals and graphene oxide. The test zone is treated with human serum and incubated, a redox indicator is subsequently added, and the process is complete. This sensor was able to detect COVID-19 in a couple of patients, and the results were comparable to that of commercial ELISA. The responses are monitored with square-wave voltammetry [141,142,143]. In other electrochemical detection applications, it was demonstrated that SARS-CoV-2 can be detected in saliva. The assay could successfully detect both S1 protein and the nucleocapsid protein using magnetic beads (MB). The sensing mechanism relies on the formulation of COVID-19 antibody–modified MB, that captures the analyte. The beads are labeled with alkaline phosphate enzymes and are deposited on a carbon-based working electrode and the response is detected using pulse voltammetry using a potentiostat. The response of the assay revealed a sensitivity for S protein of 6.5 plaque-forming units per milliliter (pfu/mL). Another research group reported eCovSens, an antigenic COVID-19 sensor that detects the S1 protein on fluorine-doped tin oxide decorated with Au NPs or screen-printed carbon electrodes. A detection limit of 80 femtoMolar (fM) and 120 fM for the S1 protein on the FTO and SPCE, respectively, was achieved in saliva in 30 s [144,145,146,147].

#### 3.3.4. Graphene Field Effect Transistor

**Figure 8:**
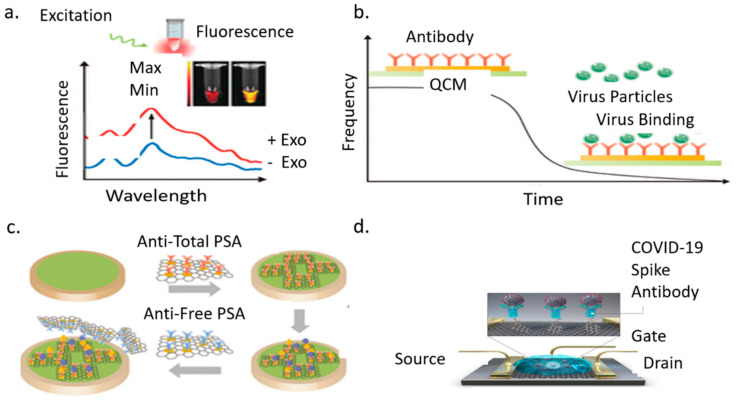
Types of biomarker sensors: (**a**). Optical biosensor; (**b**). Piezoelectric biosensor; (**c**). Electrochemical biosensor, (**d**). Graphene field-effect transistor.

A field-effect transistor (FET) based biosensing device has several advantages, including the ability to make very sensitive and instantaneous measurements using small amounts of analytes for detecting COVID-19 in clinical samples. The sensor uses graphene sheets coated with a specific antibody immobilized against SARS-CoV-2 spike protein [148,149,150,151]. Graphene is selected because of its high electron mobility, electrical and thermal conductivity, and mechanical strength. The antibody is immobilized through coupling agent 1-pyrenebutyric acid N-hydroxysuccinimide ester (PBASE), which acted as a probe linker. FET consists of three terminals: source, gate, and drain. Bioreceptors are immobilized on the gate terminal. The terminal is connected to both the source and drain electrodes [152,153,154,155]. The sensor’s performance is tested using an antigen biomarker obtained from nasopharyngeal swab specimens of infected COVID-19 patients. A potential is applied to the gate upon binding with the analyte. This binding changes the conductivity through the source-drain channel, based on which molecules are detected. Therefore, FET detects pathogens through these changes in the electric field and conductivity of the surface and channels. The FET device can detect the SARS-CoV-2 spike protein at concentrations of 1 femtogram/milliliter (fg/mL) in phosphate-buffered saline and 100 fg/mL clinical transport medium. In addition, the FET sensor successfully detected SARS-CoV-2 in culture medium limit of detection (LOD): 1.6 × 10^1^ pfu/mL) and clinical samples (LOD: 2.42 × 10^2^ copies/mL). The drawback of this technology is the baseline drift in an aqueous environment. This limits their ability to respond to target molecules [156,157,158]. Figure 8 presents the COVID-19 biomarker sensors. Figure 8a shows an optical biosensor transducer that can detect the analyte or pathogen as a measured change in fluorescence, absorption, or reflectance performance of the sensing material. The fluorescence spectra of a semiconducting polyelectrolyte nanocomplex with and without exosomes as involved in pathogenesis including neurodegenerative diseases viral/bacterial infection and cancer. The strength of the color change usually red or yellow will determine if higher concentrations of the disease are present. This technique takes advantage of the color change of the sensing material when its size or concentration changes due to interaction with analyte or pathogen. Figure 8b Piezoelectric Biosensor Quartz Crystal Microbalance (QCM) shows a change in mass on the surface of the crystal results in a proportional change in frequency this is detected by the unique interaction between the antibody and antigen. Figure 8c shows an electrochemical sensor used to detect prostate cancer. This technology is based on the responses of square-wave voltammetry of the sensor as well as the various concentrations of 0, 4, 6, 8, and 10 ng/mL of total prostate-specific antigen (PSA). Antitotal PSA antibody is attached to the surface of the working electrode for antigen capture. The resulting pairing of the complex forms a sandwich-like system with the AntiFree PSA antibody. These new technologies have potential applications to COVID-19 detection [159,160]. Figure 8d is the Graphene Field Effect Transistor described in the above section [161].

#### 3.3.5. Lateral Flow Assay

**Figure 9:**
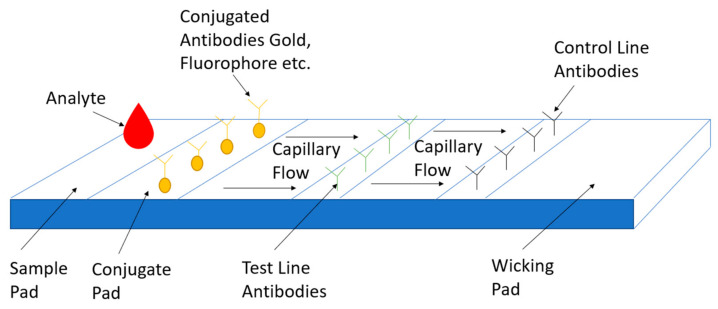
COVID-19 Point of Care Detection Lateral Flow Assay.

Lateral flow assay or (LFA) is a paper-based platform for the detection and quantification of analytes in complex mixtures, where the sample is placed on a test device, and the results are displayed within 5–30 min. Pregnancy tests are a typical example of this detection platform. The idea behind the LFA is easy and makes it a superior technology for POC applications. Capillary action moves the analyte across multiple strip zones where biomarkers are immobilized for proper interaction [162,163]. The sample pad ensures that the analyte present will bind to all reagents on the membrane. The sample moves through the conjugate pad, containing antibodies or antigens specific to the target analyte, and is conjugated to either colored or fluorescent particles colloidal gold or latex microspheres [164,165]. The complex then moves along the strip with all particles bound into the detection zone. Then it will interact with each test line to confirm results. The test line will indicate if the person is infected or not, while the control line indicates proper liquid flow through the strip. Finally, the test uses an absorbent pad to wick the excess reagents and prevent the backflow of the liquid. These tests can expand with additional test lines for multiple biomarker detection. Other applications involve semi-quantitative assays, where an increasing number of lines appearing on the strip is directly proportional to the concentration of the analyte. During COVID-19 testing, recombinant spike protein antigen reagents that specifically bind to SARS-CoV-2 antibodies (IgM and/or IgG), are bound to colloidal gold and sprayed on conjugation pads. The sample is applied to the test wells, antibody and labeled antigen complexes are formed and travel up the strip. The labeled gold colorimetric reagent forms a visible red/pink line. The presence of anti-SARS-CoV-2 IgM and/or IgG will be indicated by a visible red/pink test line (T) in the IgM and IgG result windows. Anti-SARS-CoV2 IgM antibodies are bound on the IgM line, and anti-SARS-CoV-2 IgG antibodies are bound to the IgG line. New research has identified ways to the detection sensitivity using magnetic particles such as nano-gold microspheres, or immune-nanoparticles will reduce the detection limits to at least 0.1 ng/mL [166,167]. Another way to increase assay sensitivity is by using a laser or light-emitting diode (LED). This can certainly amplify signals. Combining the use of colloidal gold nanoparticles and oligonucleotides for the simultaneous detection of antigens and antibodies is another option or the use of two conjugate pads for the simultaneous detection of two proteins. The LFA has a few drawbacks. They provide no quantitative results, low sensitivity, and the test compared to other sensing platforms is not as quick [168]. Figure 9 shows a typical LFA used for COVID-19 detection adapted from [169].

### 3.4. Imaging Detection of COVID-19

#### 3.4.1. Ultrasound Detection

Ultrasound imaging uses a probe to both emit and receive sound waves as they propagate through the body. These waves travel to a target site or area being examined until they hit a boundary between tissues, fluid, or bone [170]. Some waves will be reflected before others and based on the speed, direction and distance travel an image can be made of the area. Lung ultrasound in COVID-19 detection helps physicians to differentiate between pneumonia and dyspnea in potential COVID-19 positive cases. Visible observations of an ultrasound for a COVID-19 infected patient reveal B lines that give the appearance of a shining white lung. Irregularity of the pleural line, sub-pleural pulmonary consolidations and poor blood flow also occur in bilateral patchy clusters and are mainly visible in the posterior and inferior areas [171,172,173,174]. The use of ultrasound has many advantages. This includes use in point of care applications and low cost. It can be extended to a patient’s residence or at their bedside reducing the demand to transport the patient. This method is also noninvasive and eliminates the patient’s exposure to X-rays. Lung ultrasound score (LUS) can be used as a classification tool to determine lung deterioration severity based on patterning of pleural lines. Disadvantages are the quality of the imaging compared to both X-ray and Computerized Tomography scanning [175].

#### 3.4.2. X-ray Detection

X-ray detection is another alternative to COVID-19 detection. X-ray stands for Energetic High-Frequency Electromagnetic Radiation. An electromagnetic beam passes through the body and body tissues and bones absorb or block the beam at different densities [176]. This creates a shadow that is picked up by a sensor. Chest X-ray works on this basic principle. During COVID-19 detection, a scan of the chest is completed and, since lungs are filled with air, the X-ray will appear black. Any fluid buildup will appear gray and can be seen on the X-ray. This gradation depends on the water density. Therefore, fluid buildup in the lungs gives off a grayish color [177,178,179,180]. Radiography examination is a reliable method for detecting COVID-19. In many countries, Computerized Tomography (CT) imaging is scarce due to its high cost. X-ray offers an alternative when viral test supplies are low. It can also help in ranking the severity of patients with the disease to provide aid to the most critical patients first. Drawbacks with this method include a lack of visual indicators. Some tests are very subtle and inconclusive. Computer-aided diagnostic systems can help to increase the accuracy of detection such as COVID-net, which uses Artificial Intelligence tailored for the detection of COVID-19 cases from Chest X-ray Images [181].

#### 3.4.3. Computerized Tomography Scan-CT Scan Detection

**Figure 10:**
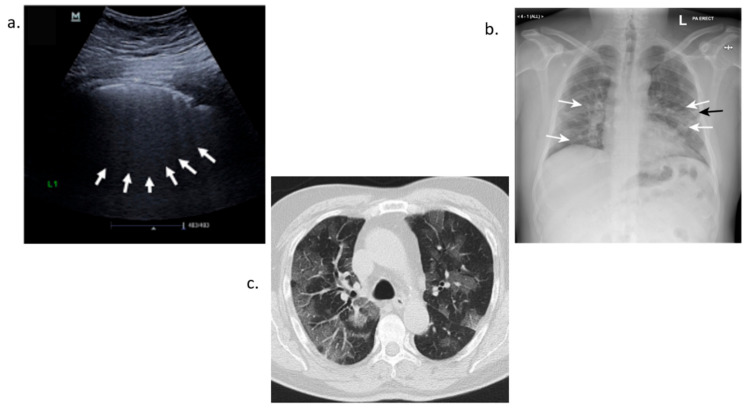
COVID-19 imaging detection (**a**). Ultrasound detection (**b**). X-ray detection (**c**). Computerized tomography scan.

Chest CT scans take X-ray measurements at different angles across a patient’s chest to produce cross-sectional images. These images are analyzed by radiologists to look for abnormal features such as ground-glass opacities or consolidations of the lungs that can lead to a diagnosis. The scans can also reveal bilateral or peripheral lesions [182,183,184,185]. Opacities can reveal several issues. Typically, normal lungs appear black in scans. However, when the air spaces are filled with fluid or the walls of the alveoli and space between the lungs begin to thicken gray areas show up in these scans. These areas are consistent with the aftermath of a cytokine storm and elevated levels of inflammatory biomarkers. Laboratory tests detected elevated levels of C-reactive protein and ferritin. Additionally, oxygen saturation was at 88% and lung auscultation showed bilateral crackles. CT scans have high sensitivity but have several limitations. They cannot be performed with patients with hypoxemia who must be mechanically ventilated for low levels of oxygen or hemodynamically unstable patients [186,187,188]. All the three methods of imaging detection discussed, CT scan, Chest X-ray and Ultrasound help with COVID-19 detection especially when RT-PCR tests are negative but the patient experiences COVID-19-like symptoms. Figure 10a shows ground glass patterns with multiple B-lines. Figure 10b highlights two areas, the white arrows for ground glass opacity and black arrows showing linear opacity. Figure 10c show a CT scan of a patient’s lungs infected with COVID-19 [189,190,191].

## 4. Future Directions

### 4.1. Challenges Currently Facing Tests on the Market

**Figure 11:**
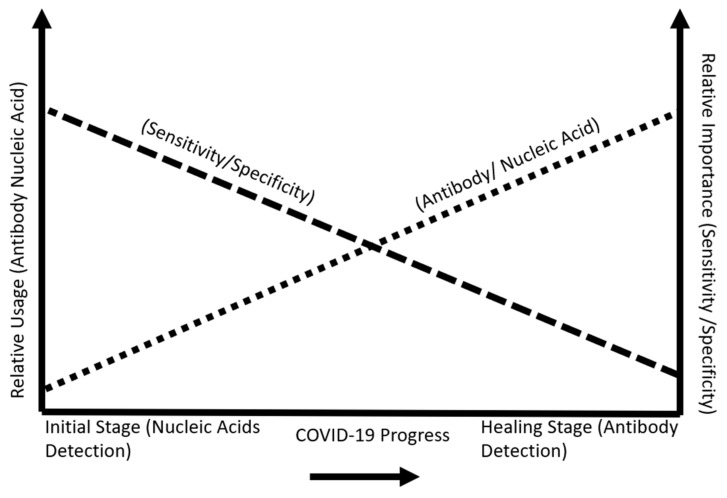
COVID-19 stage progression and importance of sensitivity and specificity.

Sensing technologies are changing the landscape of disease detection. Immuno biosensing uses a lab on a chip to measure antibody and antigen interaction. This type of technology is based on the microfluidic flow of biofluid to detect a change in capacitance. The signals are amplified and obtained from an electrode platform based on antigen and antibody interaction [192]. Other types of biosensors use carbon nanotubes to enhance the signal and sensitivity levels of biomarker disease detection [193,194]. Electrical immunosensing helps ease sensitivity detection. Many assays provide a qualitative method of biomarkers detection simply with yes or no through an observable color change. Future tests will let us know what viral concentration a person has at a given time. This will help to distinguish if a person is recovering or is infected. The POC device primarily used for COVID-19 serological testing is the lateral flow assay. This device has many drawbacks. The sensitivity is low in comparison to RT-PCR. Typically, these values are approximately 78%. This means that 22% of users will receive a false-negative due to a wide array of issues which include improper storage, incorrect swabbing, and control line missing after testing. False-negatives are worse in terms of their effect on the general population. Since patients who receive this reading will infect people unknowingly [90]. Nucleic acid amplification testing, for example, the RT-PCR test, has limitations in sensing technology as they cannot detect a low viral load of RNA in samples. As new sensing technologies develop, their LOD levels will increase. Viral loads differ in concentration values across patients. The least sensitive assays typically detect high viral loads among patients classified as (spreaders). However, low viral loads amongst patients can be near or even below the LOD of many types of assays. LOD is the lowest level concentration of a target detected in 95% or more repeated measurements. Sensitivity importance is crucial in the early detection stages of symptom development of COVID-19. Specificity is important during the recovery period after contracting the virus. Figure 11 shows this relationship [13].

### 4.2. Cross-Reactivity between Different Types of Coronaviruses

The inspection of structural relationships between SARS-COV and SARS-CoV-2 is comparable in the N gene. At six regions, there are differences in the viruses, partial coding sequences of ORF1a/b (448 nucleotides (nt), 55 nt and 278 nt, respectively), S gene (315 nt and 80 nt), and the coding sequence of the orf7b and orf8 genes (214 nt). The envelope, membrane, or accessory proteins p6 and 8b, nsp7, and nsp13 have no change in amino acid sequences. It is essential to limit regions with high similarities in a detection assay since it would be difficult to determine which type of coronavirus someone has contracted. The S1 region of the virus is divided into two regions N terminal domain (NTD) and the C terminal domain (CTD), both of which can act as the RBD (receptor-binding domain). The NTD has the lowest percent identity when comparing SARS-CoV-2 with other types of coronaviruses. The following percentages were recorded: 52.55% identity with SARS-COV, 21.67% with MERS-COV, 21.49% with HCOV-HKU1, 24% for HCOV-229E, 21% for HCOV-NL63, and 20.26% with HCOV-OC43 [195]. Therefore, the NTD region of the SARS-CoV-2 gene is the best region to target to help minimize cross-reactivity. Another approach to limit cross-reactivity would be to use multiple biomarkers with specific regions of distinction to increase SARS-CoV-2 detection.

### 4.3. Multiple Biomarkers Used for COVID-19 Detection

In many COVID-19 patients, seropositivity for a combination of antibodies was key in identifying infected patients. Some COVID-19 patients had earlier detection for the anti-receptor binding domain (RBD) than for anti-nucleocapsid (NP) for IgM and IgG. Other patients only had an anti-spike or anti-Nucleocapsid antibody seroconversion. The combination of antibodies for detection assays is a very effective strategy in early detection and screening. In one study, the combined detection of N and S specific IgM and IgG could identify up to 75% of SARS-CoV-2 infected patients in the first week [196,197].

## 5. Conclusions

This paper focuses on biomarkers for COVID-19 detection. The biomarkers best suited for detection purposes were the S protein, specifically the RBD region. Many human coronaviruses have similarities, and care should be taken not to select biomarkers prone to cross-reactivity. The IgG antibody can be detected in serum the longest, while IgA and IgM are detected earlier after symptoms develop. The S-IgG, antigen and antibody pairing complex is vital in fighting the virus. As new nanotechnology sensing capabilities increase, patients with low viral loads can be detected. The future of COVID-19 detection should be faster, reliable, simple to use, and readily available.

## 6. Patents

There are no patents that resulted from the work reported in the manuscript.

## Figures and Tables

**Table 1:** COVID-19 inflammatory biomarkers: normal vs. infected levels.

Biomarkers	Normal Patient	Infected Patient
Serum Ferritin	15.0–150.0 (ng/mL)	452.9–1451.6 (ng/mL)
C reactive protein	0.0–1.0 (ng/mL)	20.9–103.2 (ng/mL)
Interleukin 2R	223.0–710.0 (U/mL)	528.5–1136.3 (U/mL)
Cytokines (IL-6)	0.0–7.0 (pg/mL)	7.9 (pg/mL)
D-Dimer	0–0.243 (µg/mL)	0.5 (µg/mL)
Serum Amyloid A (SAA)	0–10 (mg/mL)	108.4 (mg/mL)

## Data Availability

Not applicable.

## Abbreviations

MDPI	Multidisciplinary Digital Publishing Institute
DOAJ	Directory of open access journals
TLA	Three-letter acronym
LD	Linear dichroism
RT-PCR	Reverse Transcription-Polymerase Chain Reaction
FET	Field Effect Transistor
WHO	World Health Organization
SARS-CoV-2	Severe Acute Respiratory Syndrome 2
MERS-CoV	Middle East Respiratory Syndrome
2019-nCoV	COVID-19
ACE2	Angiotensin Converting Enzyme
TMPRSS2	Transmembrane serine protease 2
DDP4	Dipeptidyl peptidase-4
EUA	Emergency Use Authorization
USD	United States Dollar
POC	Point-Of-Care
N	Nucleocapsid
S	Spike
Ig	Immunoglobulin
IL	Interleukin
ssRNA	single-strand Ribonucleic Acid
kbp	kilobase pairs
ORF	Open reading frame
NSP	Non-Structural Protein
RdRp	RNA dependent RNA Polymerase
NTD	Amino Group Terminal Domain
CTD	Carboxyl Group Terminal Domain
nM	nanoMolar
Abs	Antibodies
U/mL	Units per milliliter
IFN	Interferon
EGF	Epidermal Growth Factor
PDGF	Platelet Derived Growth Factor
VEGF	Vascular endothelial Growth Factor
CXCL	Chemokine
TNF	Tumor Necrosis Factor
ng/mL	nanograms per milliliter
pg/mL	picograms per milliliter
µg/mL	microgram per milliliter
fg/mL	femtogram per milliliter
DNA	Deoxyribonucleic acid
cDNA	Complimentary DNA
NP	Nucleocapsid Protein
SP	Spike Protein
NAAT	Nucleic Acid Amplification Technique
LAMP	Loop-mediated isothermal amplification
CRISPR	Clustered Regularly Interspaced Short Palindromic Repeats
DETECTR	DNA Endonuclease Targeted CRISPR Trans Reporter
TIR	Internal Reflection
RI	Refractive Index
RIU	Refractive Index Units
SPR	Surface Plasmon Resonance
LSPR	Localized Surface Plasmon Resonance
AuNI	Gold Nano Island
PPT	Plasmonic Photothermal
PQC	Piezoelectric Quartz Crystal
MB	Magnetic Beads
pfu/mL	plaque forming units
AuNPs	Gold Nanoparticles
fM	femtoMolar
FTO	Fluorine Doped Tin Oxide
SPCE	Screen Printed Carbon Electrodes
AC	Alternating Current
FET	Field Effect Transistor
PBASE	1-pyrenebutanoic acid succinimidyl ester
LOD	Limit of Detection
LFA	Lateral Flow Assay
LED	Led Emitting Diode
LUS	Lung Ultrasound Score
Xray	Energetic High-Frequency Electromagnetic Radiation
CT	Computerized Tomography
nt	nucleotide
